# The pleiotropic effects of statins: a comprehensive exploration of neurovascular unit modulation and blood–brain barrier protection

**DOI:** 10.1186/s10020-024-01025-0

**Published:** 2024-12-20

**Authors:** Jia-Cheng Liu, Shuang-Yin Lei, Dian-Hui Zhang, Qian-Yan He, Ying-Ying Sun, Hong-Jing Zhu, Yang Qu, Sheng-Yu Zhou, Yi Yang, Chao Li, Zhen-Ni Guo

**Affiliations:** 1 Stroke Center, Department of Neurology, The First Hospital of Jilin University, Xinmin Street 1#, Changchun, 130021 China; 2 Jilin Provincial Key Laboratory of Cerebrovascular Disease, Xinmin Street 1#, Changchun, 130021 China; 3 Neuroscience Research Center, The First Hospital of Jilin University, Xinmin Street 1#, Changchun, 130021 China

**Keywords:** Statins, Pleiotropic effects, Neurovascular unit, Blood–brain barrier, Ischemic stroke

## Abstract

The blood–brain barrier (BBB) is the most central component of the neurovascular unit (NVU) and is crucial for the maintenance of the internal environment of the central nervous system and the regulation of homeostasis. A multitude of neuroprotective agents have been developed to exert neuroprotective effects and improve the prognosis of patients with ischemic stroke. These agents have been designed to maintain integrity and promote BBB repair. Statins are widely used as pharmacological agents for the treatment and prevention of ischemic stroke, making them a cornerstone in the pharmacological armamentarium for this condition. The primary mechanism of action is the reduction of serum cholesterol through the inhibition of 3-hydroxy-3-methylglutaryl-coenzyme A (HMG-CoA) reductase, which results in a decrease in low-density lipoprotein cholesterol (LDL-C) and an increase in cholesterol clearance. Nevertheless, basic and clinical research has indicated that statins may exert additional pleiotropic effects beyond LDL-C reduction. Previous studies on ischemic stroke have demonstrated that statins can enhance neurological function, reduce inflammation, and promote angiogenic and synaptic processes following ischemic stroke. The BBB has been increasingly recognized for its role in the development and progression of ischemic stroke. Statins have also been found to play a potential BBB protective role by affecting members of the NVU. This review aimed to provide a comprehensive theoretical basis for the clinical application of statins by systematically detailing how statins influence the BBB, particularly focusing on the regulation of the function of each member of the NVU.

**Graphical Abstract:**

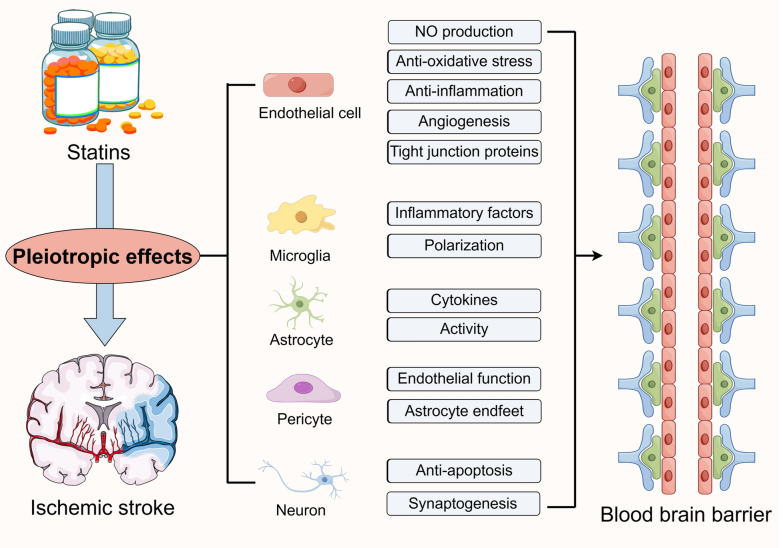

## Introduction

It is essential to regulate cerebral blood flow (CBF), oxygen transport, and energy metabolite translocation, which are primarily mediated by the neurovascular unit (NVU), to maintain the normal physiological functions of the brain. These functions include structural and functional connectivity, information transmission, and processing (Zhao et al. 2015). The blood–brain barrier (BBB) is a fundamental structural element of the NVU. The BBB separates the central nervous system (CNS) from the peripheral system and maintains a stable environment within the CNS. The BBB serves as a pivotal link between the CNS and the peripheral system. It regulates the passage of nutrients, metabolites, and other essential molecules into and out of the CNS (Hawkins and Davis 2005). Several CNS diseases can cause the breakdown of the BBB. Among these, ischemic stroke, which has the highest incidence among cerebrovascular diseases, is closely associated with BBB in its pathogenesis and pathophysiological changes (Zlokovic 2008). Following the onset of ischemic stroke, a multitude of pathophysiological alterations mediate the onset of BBB dysfunction. These include enhancement of the local inflammatory response (Candelario-Jalil et al. 2022), activation of oxidative and nitrosative stress (Zhou et al. 2021), alterations in adhesion molecules and leukocyte infiltration (Yilmaz and Granger 2008), and an increase in matrix metalloproteinases and basement membrane disruption (Rosenberg et al. 1998). Following the disruption of the BBB, blood-borne components can infiltrate the brain parenchyma, resulting in cerebral edema (Keaney and Campbell 2015). Leukocyte infiltration exacerbates the inflammatory response and extent of brain injury (Candelario-Jalil et al. 2022). Consequently, alleviation of BBB dysfunction in the treatment of ischemic stroke has become a focus of both basic and clinical research.

**Fig. 1:**
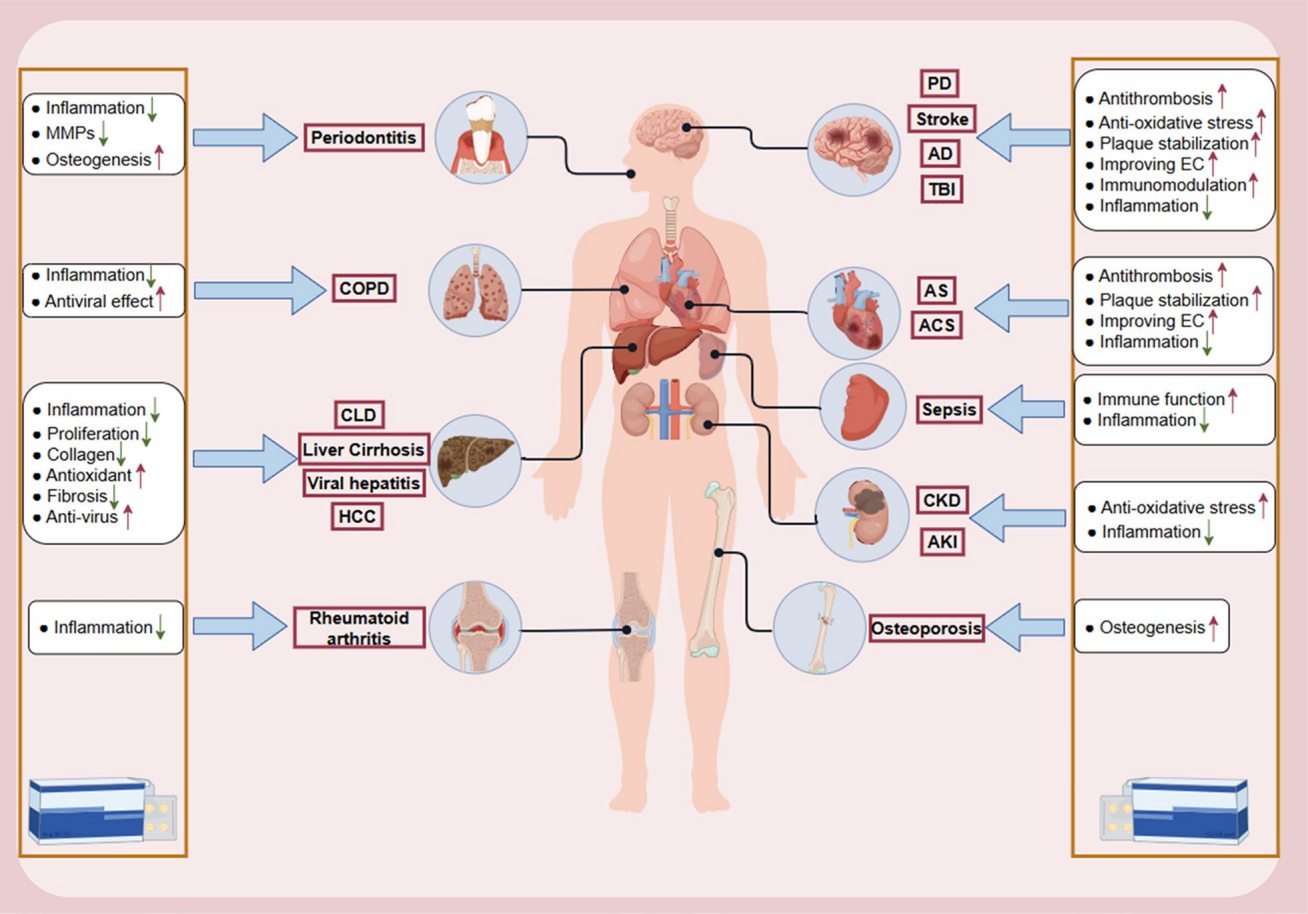
Pleiotropic effects and mechanisms of statins in various organs or systems (McCarey et al. 2004; Ray and Cannon 2007; Yanuck et al. 2012; Oesterle et al. 2017; Imprialos et al. 2018; Kong et al. 2018; Lin et al. 2018; Verdoodt et al. 2018; Amariei and Reed 2019; Gupta et al. 2019). COPD, chronic obstructive pulmonary disease; CLD, chronic liver disease; HCC, hepatocellular carcinoma; PD, Parkinson’s disease; AD, Alzheimer’s disease; TBI, Traumatic brain injury; AS, atherosclerosis; ACS, acute coronary syndrome; CKD, chronic kidney disease; AKI, acute kidney injury; EC, endothelial cell

Statins are a class of pharmaceutical agents that act as inhibitors of 3-hydroxy-3-methylglutaryl-coenzyme A (HMG-CoA) reductase, which are commonly used to reduce lipid levels in patients with cardiovascular disease (Christophe et al. 2020). The Heart Protection Study (HPS) demonstrated that simvastatin has a significant impact on reducing mortality and morbidity associated with coronary heart disease and stroke (Hamilton-Craig 2002). In accordance with the clinical care guidelines for cerebrovascular disease in China (Liu et al. 2023) and the guidelines for the prevention of stroke from the American Heart Association/American Stroke Association (Kleindorfer et al. 2021), lipid-lowering therapy with statin is recommended for patients with both non-cardioembolic ischemic stroke and transient ischemic attack, and this therapy is a class I recommendation with level A evidence. This suggests that statins are very common in ischemic stroke treatment and prevention. Nevertheless, numerous randomized controlled trials have demonstrated that statins are more efficacious and beneficial than lipid-lowering therapies without statins or with other lipid-lowering drugs, even in individuals with normal cholesterol levels (Long-Term Intervention with Pravastatin in Ischaemic Disease (LIPID) Study Group 1998; Collins et al. 2004; Ní Chróinín et al. 2011; Flint et al. 2012; Kitagawa et al. 2017). These findings indicate that statins have a broader function than reducing cholesterol levels. Previous studies on ischemic stroke have demonstrated that statins enhance neurological function, augment CBF (Cimino et al. 2007), reduce the impact of inflammation and post-traumatic perfusion deficits (Wang et al. 2008), and safeguard blood vessels while fostering angiogenic and synaptic processes following ischemic stroke (Zhang et al. 2005b). The phenomenon of statins having an impact beyond lowering cholesterol production is known as pleiotropy (German and Liao 2023). Furthermore, the pleiotropic effects of statins have been demonstrated in other systemic diseases. A summary of the evidence for the pleiotropic effects of statins in other systems is presented in Fig. 1 and Table 1.

**Table 1:** Pleiotropic effects of statins in other systems

System or organ	Disease	Type of statins	Possible pathways of pleiotropic effects	References
Cardiovascular system	Atherosclerosis	Simvastatin, atorvastatin, lovastatin, fluvastatin	• Stable atherosclerotic plaque • Anti-inflammation	Oesterle et al. (2017)
	Acute coronary syndrome	Undifferentiated	• Anti-inflammation • Improve endothelial function • Reduced platelet aggregation • Improved vascular tone • Stable atherosclerotic plaque	Ray and Cannon (2007)
Nervous system	Stroke	Rosuvastatin, simvastatin, atorvastatin, pravastatin,	• Stabilize the endothelial cell layer • Reduce oxidative stress • Promote cerebral angiogenesis • Anti-inflammation	Yanuck et al. (2012)
	Alzheimer’s disease	Undifferentiated	• Impair production of beta-amyloid proteins, apolipoprotein E, and tau fibrillization • Anti-inflammation	
	Traumatic brain injury	Rosuvastatin, simvastatin	• Preserve blood brain barrier function • Protection of neurons • Anti-inflammation	
	Parkinson’s disease	Simvastatin, lovastatin	• Decrease glial activation and oxidative stress • Anti-inflammation • Normalize striatal neurotransmitters	
Liver	Chronic Liver Disease	Atorvastatin, simvastatin	• Increased bioavailability of nitric oxide • Reduction of collagen production • Anti-inflammation • Antioxidant properties	Imprialos et al. (2018)
	Liver Cirrhosis	Atorvastatin, simvastatin	• Increased bioavailability of nitric oxide • Anti-inflammation	
	Liver disease induced by viral hepatitis	Atorvastatin, fluvastatin	• Reduction of hepatitis virus replication	
	Hepatocellular carcinoma	Atorvastatin, fluvastatin	• Inhibition of proliferation • Increased apoptosis	
Kidney	Chronic kidney disease	Rosuvastatin, atorvastatin	• Anti-inflammation • Anti-oxidant activity	Verdoodt et al. (2018)
	Acute kidney injury	Rosuvastatin, atorvastatin	• Anti-inflammation • Renal protection	
Lung	Chronic obstructive pulmonary disease	Simvastatin	• Anti-inflammation • Antiviral effect	Amariei and Reed (2019)
Spleen	Sepsis	Simvastatin	• Regulation of immune function • Anti-inflammation	(Kong et al. (2018)
Bone and joint	Osteoporosis	Rosuvastatin, atorvastatin, simvastatin	• Increase serum calcium levels • Promoting osteogenic gene expression • Increase bone density • Inhibition of osteoclast formation	Lin et al. (2018)
	Rheumatoid arthritis	Atorvastatin	• Anti-inflammation	McCarey et al. (2004)
Oral cavity	Periodontitis	Simvastatin	• Decreasing the expression of proinflammatory cytokines • Reduce matrix metalloproteinases • Promote bone formation	Gupta et al. (2019)

Several clinical investigations have demonstrated that statins have a neuroprotective function in the treatment of ischemic stroke (Long-Term Intervention with Pravastatin in Ischaemic Disease (LIPID) Study Group 1998; Collins et al. 2004; Ní Chróinín et al. 2011). These benefits are achieved by pleiotropic mechanisms and have been demonstrated to have the potential to protect the BBB in both in vivo and in vitro (Christophe et al. 2020). Nevertheless, most of these studies have concentrated on examining single cells or the overall neurological processes of the brain, leaving the mechanisms and potential advantages yet to be investigated. Consequently, the objective of this review was to summarize comprehensively the mechanisms by which statins maintain BBB integrity and attenuate BBB dysfunction in CNS diseases, particularly focusing on ischemic stroke. This can be achieved from the perspective of the NVU, offering a more systematic theoretical basis for the clinical application of statins.

### Mechanisms of pleiotropic effects of statins

**Fig.2:**
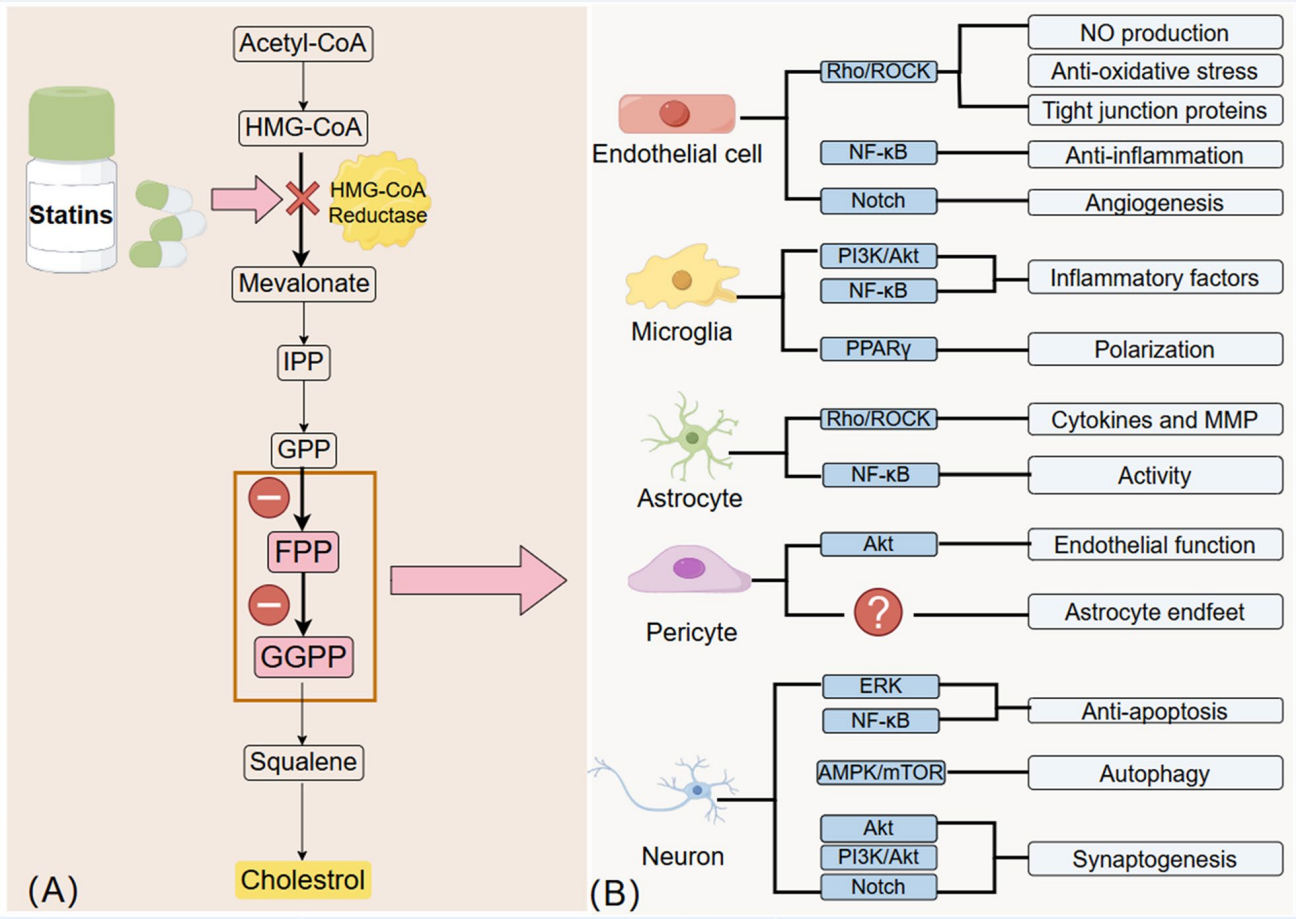
**A** Mechanism of the pleiotropic effects of statins: statins exert pleiotropic effects by inhibiting HMG-CoA reductase to reduce the production of FPP and GGPP, affecting multiple signaling pathways. **B** Pleiotropic effects of statins on individual NVU cells. Acetyl-CoA: Acetoacetyl coenzyme A; HMG-CoA: 3-hydroxy-methylglutaryl coenzyme A; IPP: isopentenyl pyrophosphate; GPP: geranyl pyrophosphate; FPP: farnesyl pyrophosphate; GGPP: geranylgeranyl pyrophosphate; ROCK: Rho-associated protein kinase; AMPK, adenosine monophosphate-activated protein kinase; ERK: extracellular regulated protein kinases; NF-κB: nuclear factor kappa-B; NVU: neurovascular unit

Statins are classified into two groups based on their lipophilicity: lipophilic statins, which include atorvastatin, simvastatin, pitavastatin, and cerivastatin and hydrophilic statins, which include rosuvastatin and pravastatin (Wood et al. 2014). Statins function primarily by competitively and selectively inhibiting HMG-CoA reductase, an enzyme that controls cholesterol production. This enzyme catalyzes the conversion of HMG-CoA to mevalonate, a cholesterol precursor (Laws et al. 2004). Statins prevent the formation of isoprenoid intermediates, farnesyl pyrophosphate (FPP) and geranylgeranyl pyrophosphate (GGPP), by inhibiting HMG-CoA reductase and decreasing mevalonate synthesis (Wang et al. 2008). Inhibition of FPP and GGPP production represents a central aspect of the pleiotropic effects of statins. FPP and GGPP are post-translational modifications of lipid-anchored heterotrimeric G proteins, which mainly comprise Ras and Rho (Van Aelst and D’Souza-Schorey 1997) (Fig. 2). Ras and Rho regulate several cellular processes, including cell proliferation, differentiation, apoptosis, and the cytoskeleton (Cho et al. 2011). The pleiotropic effects of statins have been demonstrated in several studies that have identified several signaling pathways affected by statins. These include the Rho/ROCK (Rikitake and Liao 2005), Rho/Rac1 (Ohkawara et al. 2010), TLRs/NF-κB (Dichtl et al. 2003), PI3K/Akt (Ahmadi et al. 2023), MAPKs (Ahmadi et al. 2023), ERK (Wu et al. 2013), and Notch signaling pathways (Zacharek et al. 2009).

### Mechanisms of the pleiotropic effects of statins in the NVU

**Fig. 3:**
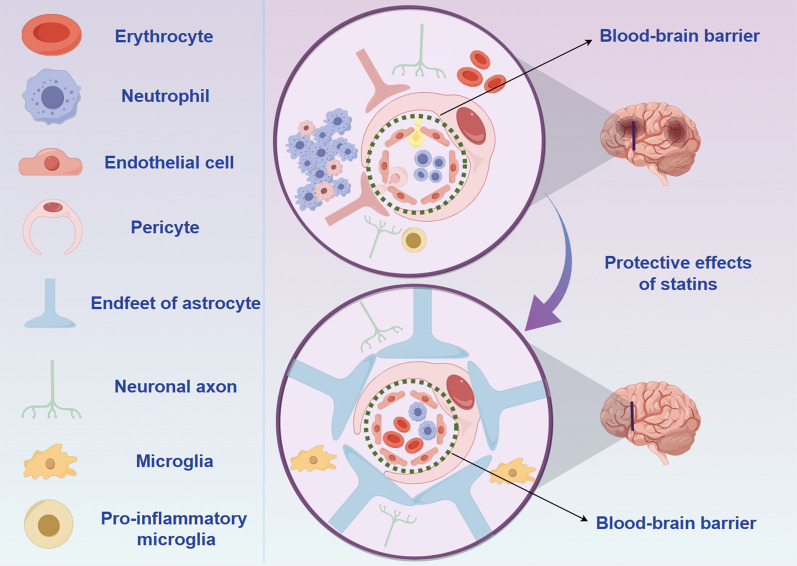
The effect of ischemic stroke on the blood–brain barrier (BBB) and the role of statins in BBB protection through the neurovascular unit (NVU). Ischemic stroke triggers endothelial cell dysfunction, resulting in the breakdown of the BBB, which allows inflammatory cells to infiltrate the vasculature and enter the central nervous system. Additionally, this condition activates microglia, driving their transformation into a pro-inflammatory state. Statins contribute to BBB repair and uphold BBB integrity by modulating the functions of various components within the NVU

The NVU is a highly complex structure composed primarily of vascular cells (endothelial cells, pericytes, and smooth muscle cells), neuroglial cells (astrocytes, oligodendrocytes, and microglia), and neurons (Zhao et al. 2015) (Fig. 3). Each member of the NVU is structurally and functionally interconnected. Consequently, any alterations in the function of a member can affect the overall function of the NVU and potentially lead to CNS disorders (Gullotta et al. 2023). Given the pivotal role of the NVU in regulating BBB function, there is growing interest in targeting the NVU for the development of novel therapeutics. Statins have been used in the treatment of CNS diseases, particularly ischemic stroke, and their pleiotropic effects have been demonstrated in multiple neurological disorders. We begin by summarizing the cellular components constituting the NVU. We then elucidate the potential mechanisms by which statins exert their pleiotropic effects on the NVU and how statins modulate the BBB through the NVU.

### Endothelial cells (ECs)

**Fig. 4:**
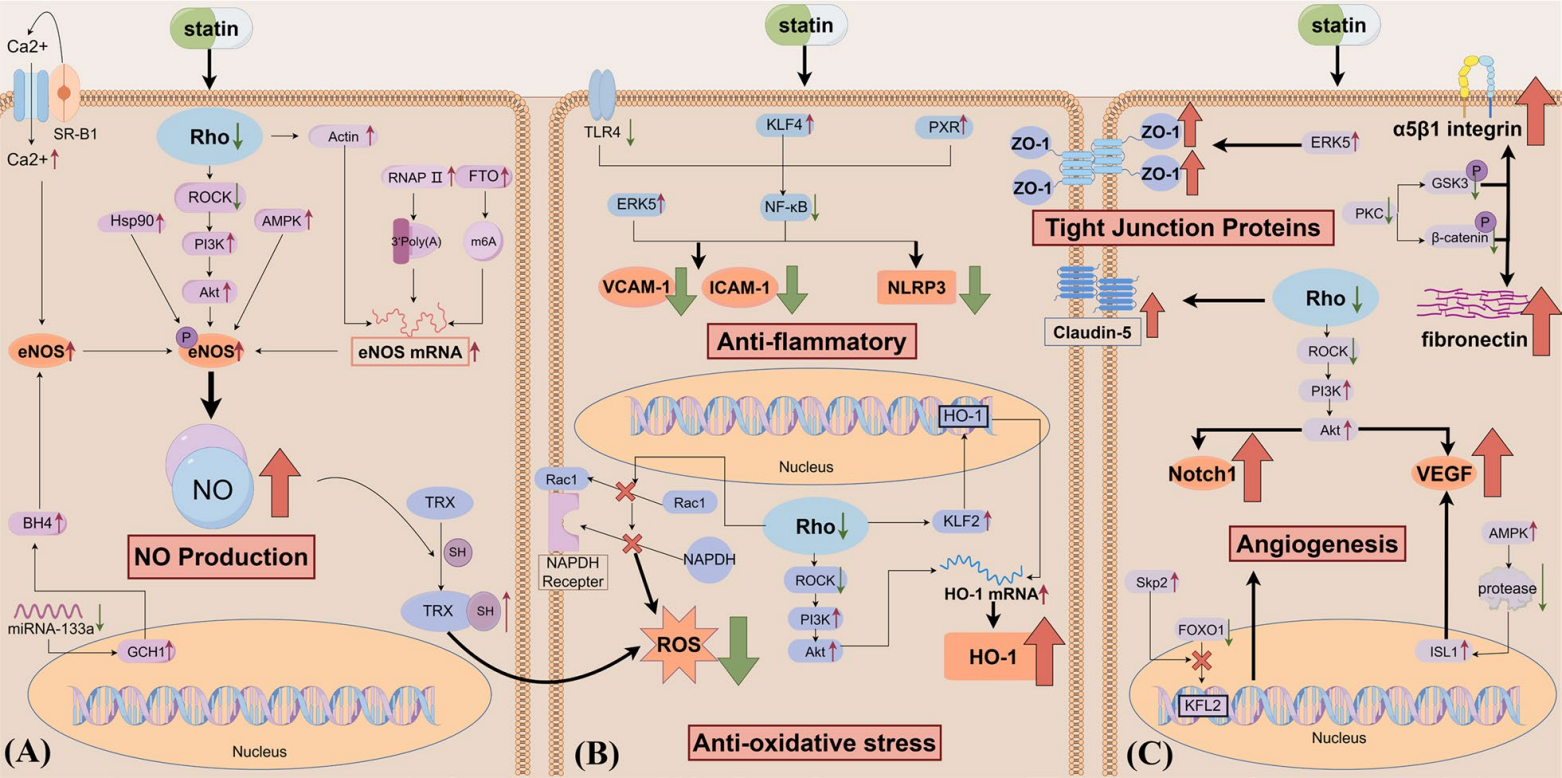
Pleiotropic effects and mechanisms of statins in cerebrovascular ECs. **A** Mechanisms by which statins improve ECs function. **B** Antioxidative stress and anti-inflammatory effects of statins in ECs. **C** Mechanisms by which statins promote angiogenesis and increase TJPs in ECs. SR-B1, scavenger receptor-B1; RNAP II, RNA polymerase II; FTO, Fat Mass and Obesity-Associated Protein; TRX, thioredoxin-1; GCH1, GTP cyclohydrolase 1; BH4, tetrahydrobiopterin; ISL1, insulin gene enhancer protein 1; HO-1, Heme oxygenase 1

It is beyond doubt that ECs represent the most crucial element of the BBB. ECs provide the structural foundation of the BBB by lacking fenestrations and expressing tight junction proteins (TJPs), creating a “physical barrier” that divides the CNS from the periphery (Zhao et al. 2015). The ability of ECs to regulate the movement of substances into and out of the CNS, which can effectively function as a "barrier" for the BBB (Westergaard and Brightman 1973), determines the role of ECs in the maintenance of CNS homeostasis (Zhao et al. 2015). Modulation of EC function is a crucial approach for maintaining BBB integrity. Several previous studies have demonstrated that statins can improve EC function (German and Liao 2023). This review summarizes the aspects related to cerebrovascular ECs and describes the mechanism of action of statins on cerebrovascular ECs. It also introduces the mechanism of statin pleiotropy in non-cerebrovascular ECs, which complements and prospects the specific mechanism of statin pleiotropic effects in the BBB (Fig. 4).

#### Improvement in endothelial function

Endothelium-produced nitric oxide (NO) is a crucial natural regulator of CBF and cerebrovascular defense mechanisms. NO has several advantageous functions in blood vessels, including vasodilation, prevention of blood clot formation, reduction of inflammation, and inhibition of cell proliferation (Sawada and Liao 2009). The conversion of NO to L-citrulline is primarily mediated by endothelial NO synthase (eNOS), which uses l-arginine as a substrate for oxidative conversion (Rudic and Sessa 1999). Following cerebral ischemia, the release of NO from ECs through eNOS can stimulate vasodilation and inhibit microvascular aggregation and adhesion, exerting a protective effect (Moro et al. 2004). eNOS reduces infarct size by regulating CBF in areas affected by ischemia. This effect is a key objective in the treatment of ischemic stroke (Dalkara et al. 1994). eNOS and NO are significant targets for regulating EC function. Statins can upregulate the expression of eNOS through multiple mechanisms at the transcriptional, protein, and genetic levels.

**Transcriptional level** Previous research has demonstrated that the activation of the Rho/ROCK pathway under hypoxic conditions can cause the dephosphorylation of eNOS, a reduction in the stability of eNOS mRNA, and ultimately, a limitation in NO production (Ming et al. 2002). The same phenomenon was observed in in vivo studies (Shin et al. 2007). Statins have been demonstrated to extend the stability of eNOS mRNA by blocking the Rho/ROCK pathway, resulting in an increase in eNOS expression (Rikitake and Liao 2005). In addition, statins have demonstrated the ability to directly enhance eNOS expression, regardless of cholesterol level. This direct enhancement is achieved in part by inhibiting the small GTPase RhoA through intracellular GGPP depletion (Sawada and Liao 2009). Statins can enhance the stability of eNOS mRNA by affecting its structure. The 3’ poly (A) tail is a crucial factor that influences mRNA stability and translation efficiency (Sachs and Wahle 1993). Statins have been linked to elevated RNA polymerase (RNAP) II phosphorylation, which alters the phosphorylation state of RNAPII in a manner that promotes mRNA 3’ polyadenylation (Kosmidou et al. 2007). Furthermore, the stability of eNOS mRNA can be influenced by statins through regulation of the actin cytoskeleton. Ou et al. (2005) demonstrated that actin influences eNOS mRNA stability and that the Rho GTPase signaling pathway plays a role in regulating the actin cytoskeleton. This suggests that statins enhance eNOS mRNA stability by inducing Rho-mediated alterations in actin cytoskeleton. Moreover, Fat Mass and Obesity-Associated Protein (FTO) have been identified as a novel mediator of the effects of statins in ECs. FTO has been identified as a regulator of m6A modification, which affects mRNA stability. Atorvastatin can enhance eNOS expression and protect ECs by reducing FTO protein levels (Mo et al. 2022). Besides directly affecting mRNA structure, statins may also affect the post-transcriptional regulation of eNOS through miRNA-related pathways. miRNA-221/222 has been associated with the reduction of eNOS mRNA and NO release in ECs (Suárez et al. 2007). Cerda et al. (2015) demonstrated that atorvastatin reduced the expression of miRNA-221 and miRNA-222 in ECs. However, the predictive analysis of bioinformatics tools indicated there was no target region for miRNA-221/222 in eNOS mRNA molecules that generate mRNA-miRNA interactions. Furthermore, available studies do not provide evidence that miRNA-221/222 is associated with eNOS regulation or NO release. Further experiments are required to elucidate the relationship between statin-induced miRNA-221/222 downregulation and eNOS expression.

These studies have demonstrated that statins affect mRNA homeostasis of eNOS gene post-transcription, which upregulates the expression of eNOS and ultimately promotes the production and release of NO in ECs. Whether statins regulate eNOS expression at the gene level has been a subject of considerable debate. Laufs and Rikitake et al. demonstrated that RhoA inhibitors, ROCK inhibitors, and actin polymerization inhibitors enhance eNOS mRNA in cultured ECs by extending the half-life of eNOS mRNA rather than affecting eNOS gene transcription (Laufs et al. 2000; Rikitake et al. 2005). Consequently, further studies must ascertain whether statins can affect eNOS gene of eNOS.

**Protein level** At the protein level, statins affect NO production by regulating eNOS protein expression and activation. The phosphorylation of eNOS is a crucial mechanism for regulating eNOS protein activity. Ser-617, Ser-635, and Ser-1179 are common phosphorylation sites in eNOS proteins, as previously demonstrated (Sawada and Liao 2009). A study using the middle cerebral artery occlusion (MCAO) model demonstrated that transgenic mice expressing the phosphorylated (S1179D) form of eNOS displayed enhanced vascular reactivity, reduced occurrence of severe stroke, and improved CBF compared to mice expressing the non-phosphorylated (S1179A) form of eNOS (Atochin et al. 2007). Statins inhibit the Rho/ROCK pathway, which activates the PI3K/Akt pathway. This results in rapid phosphorylation of eNOS proteins and the subsequent rapid release of NO (Ahmadi et al. 2023). This indicates that statins primarily affect the phosphorylation of eNOS proteins via the PI3K/Akt pathway. Besides the Rho/ROCK pathway, statins regulate the PI3K/Akt pathway through other pathways. Brouet et al. (2001) demonstrated that atorvastatin-induced phosphorylation of eNOS Ser1177 depends on heat shock protein 90. Heat shock protein 90 facilitates the incorporation of Akt into the eNOS complex, increasing eNOS protein phosphorylation (Brouet et al. 2001). Adenosine monophosphate-activated protein kinase (AMPK) also plays a significant role in eNOS phosphorylation. Previous studies have demonstrated that AMPK can activate eNOS and enhance NO production, particularly in EC, by phosphorylating Ser-1177/1179 of eNOS (Chen et al. 2003a). In a study conducted by Sun et al. (2006), atorvastatin was found to enhance eNOS activation in EC by promoting the phosphorylation of AMPK at Thr-172. This effect could be inhibited by compound C, an AMPK antagonist. Additionally, they showed that the activation of eNOS by AMPK is relatively independent of the PI3K/Akt pathway. Further investigations revealed that simvastatin promotes Ca2⁺ influx in EC through the activation of transient receptor potential vanilloid type 1 (TRPV1), subsequently activating TRPV1-TRP ankyrin 1 signaling, which leads to the phosphorylation of calmodulin-dependent protein kinase II (CaMKII), a key component of the PI3K/Akt pathway. Consequently, this process facilitates the formation of the TRPV1-CaMKII-AMPK-eNOS complex, ultimately activating eNOS and increasing NO bioavailability (Su et al. 2014).

For eNOS to produce NO, it must be filled with tetrahydrobiopterin (BH4). GTP cyclohydrolase 1 (GCH1), an enzyme that controls BH4 production, is essential for the proper functioning of eNOS (Du et al. 2008). A study has demonstrated that lovastatin can reduce aberrant miRNA-133a levels and enhance GCH1 gene expression in ECs, which helps restore BH4 levels and eNOS recoupling, improving endothelial dysfunction (Li et al. 2016). Currently, there is a lack of research exploring the alterations in BH4 levels in cerebrovascular ECs following the onset of ischemic stroke. BH4 has been extensively discussed in the context of myocardial ischemia/reperfusion, with studies indicating that elevating BH4 in ECs is beneficial in this scenario (Xie et al. 2015b). However, studies pertaining to ischemic stroke have yielded contrasting findings. While BH4 treatment was shown to enhance NOS and NO levels in brain tissue, it also resulted in a larger volume of cerebral infarcts in the BH4-treated group (Tang and Zheng 2011). This phenomenon was attributed by this author to the presence of other NOS subtypes in brain tissue, specifically neuronal NOS (nNOS) and inducible NOS (iNOS). It is widely acknowledged that n/iNOSs generate excessive levels of NO, leading to apoptosis and/or necrosis of neurons, whereas eNOS produces modest amounts of NO, improving neurological function by augmenting CBF (Wu et al. 2023). Notably, Endres et al. (1998) observed that statins increased eNOS levels in brain tissue after ischemia–reperfusion without affecting nNOS and iNOS levels. This suggests that statins exhibit a tissue-specific or selective effect on eNOS expression. The possible involvement of statins in specifically regulating BH4 levels in ECs through certain pathways remains an intriguing area for future investigation.

Kruppel-like factor 2 (KLF2) is also a key regulator of EC homeostasis. KLF2 facilitates the accumulation of eNOS in ECs through post-transcriptional mechanisms, protein synthesis, and stability, resulting in elevated eNOS levels (Sen-Banerjee et al. 2005). In ischemic stroke, KLF2 has recently emerged as a novel therapeutic target, particularly significant in the regulation of BBB function (Zhang and Li 2020; Li et al. 2024). In vitro experiments revealed that oxygen–glucose deprivation/reoxygenation (OGD/R)-induced injury significantly reduced KLF2 levels in cerebrovascular ECs. In contrast, enhanced KLF2 activity led to increased eNOS expression, thereby alleviating EC dysfunction (Zhang and Li 2020; Li et al. 2024). These findings suggest that statins' capacity to improve KLF2-mediated endothelial impairments during ischemic stroke is a promising area for further exploration. Shi et al.’s work (Shi et al. 2013) is currently advancing in this direction. We foresee a more comprehensive and robust understanding of the pleiotropic effects of statins in neuroprotection, as research on statins and KLF2 in ischemic stroke continues to expand.

**Other ways** Statins also modulate eNOS activation, independent of their cholesterol-lowering and pleiotropic effects. In clinical trials, elevation in circulating endothelial progenitor cells (EPCs) following acute ischemic stroke has been linked to positive functional outcomes, decreased infarct size, and neurological enhancement (Chu et al. 2008). After 24 h of atorvastatin treatment, serum levels of vascular endothelial growth factor (VEGF), active MMP-9, and NO were considerably elevated, and these substances were also linked to high levels of circulating EPCs (Sobrino et al. 2012). Statins have been demonstrated to enhance the expression of angiogenic factors, including eNOS and VEGF, in ECs, facilitating the mobilization and growth of EPCs (Nakata et al. 2007). These findings indicate the existence of direct eNOS activation pathways for statins that result in NO production.

Interestingly, Datar et al. (2010) demonstrated in vitro that lovastatin and pravastatin both can rapidly and directly activate eNOS through a specific EC receptor, scavenger receptor-B1 (SR-B1), which is associated with extracellular calcium. As previously mentioned, statin-induced promotion of calcium (Ca2 +) influx also activates AMPK, which subsequently enhances eNOS expression and NO production (Su et al. 2014). Previous studies have indicated that the Ca2 + -dependent kinase signaling pathway is one of the primary routes through which statins (including lovastatin, atorvastatin, pravastatin, and simvastatin) enhance eNOS activity and NO bioavailability (Chen et al. 2024). However, a key mechanism involved in ischemic stroke is cellular calcium overload. During ischemia, cells fail to generate sufficient energy to maintain proper function of Ca2 + -related channels and transporters due to blood and oxygen deprivation, leading to increased intracellular calcium concentration (Maida et al. 2024). This rise in intracellular calcium not only promotes the release of calcium from mitochondria and other cellular stores, but also results in mitochondrial dysfunction and accelerated cell death (Suzuki et al. 2022). This raises critical questions: does statin still promote eNOS expression via Ca2 + influx-related pathways under ischemic conditions, and does activated Ca2 + exacerbate endothelial cell calcium overload?

Arnould et al. (1992) observed that even after 2 h of hypoxia following oxygen–glucose deprivation (OGD) treatment of endothelial cells in vitro, the Ca2 + concentration remained significantly below the threshold for triggering cell death. They further suggested that this hypoxia-induced increase in Ca2 + activates Ca2 + -related signaling pathways, thereby altering endothelial cell function. Recent studies have also shown an elevated activity of calmodulin (CaM) in ECs and brain tissue of a rat model of ischemic stroke, which mediates post-ischemia cellular calcium overload (Zhang et al. 2024). Increased intracellular calcium modifies the activities of enzymes such as Ca2 + -CaM-dependent protein kinase II, protein kinase C, phospholipase A2, protease, nitric oxide synthase, calmodulin phosphatase, and nucleic acid endonuclease, leading to changes in cellular function. While Ca2 + influx promotes eNOS activation and NO production, existing studies suggest that calcium overload is more likely to induce oxidative stress, inflammatory factor production, and disruption of the cytoskeleton and intercellular junctions in endothelial cells (Gourdin et al. 2009). Therefore, for ECs, ischemia-induced Ca2 + influx has a predominantly negative effect. Conversely, statins continue to exert a protective role in ischemic stroke, with the upregulation of eNOS being one of the main pathways, as evidenced by various in vivo and in vitro experiments (Giannopoulos et al. 2012).

No study has yet definitively answered the aforementioned questions. However, based on the summarized mechanisms of statins’ regulation of eNOS, we hypothesize that statins’ regulation of eNOS is multifaceted rather than singular. The pleiotropic nature of statins may yield varying effects across different diseases, pathological conditions, and cell types. Determining which mechanisms and pathways are dominant and the roles they play warrants further investigation in future studies.

#### Antioxidative stress

The beneficial effects of statins under oxidative stress conditions have been demonstrated in many neuropathological studies, including ischemic stroke (Hayashi et al. 2005). Rac1, a small GTP-binding protein belonging to the Rho protein subfamily, is crucial for the formation and initiation of nicotinamide adenine dinucleotide phosphate (NADPH) oxidase (Wassmann et al. 2001). Statins can hinder Rac1 activation by preventing Rac1 translocation from the cytoplasm to the cell membrane. This inhibits NADPH oxidase activity, reduces superoxide generation, and eventually decreases cerebral infarction volume (Wagner et al. 2000). In addition, pravastatin quickly suppressed the expression of RhoA and Rac1 and decreased NADPH oxidase-dependent ROS generation in rat ECs (Ohkawara et al. 2010). An in vitro study also showed that simvastatin has a protective effect on EC barrier function, revealing that statins influence oxidative stress in ECs, mostly through Rac1 (Chen et al. 2008). Therefore, both in vivo and in vitro studies have demonstrated that statins inhibit the activation of Rac1 in ECs, leading to a decrease in NAPDH oxidase-induced ROS production, which attenuates EC oxidative stress and produces cytoprotective effects (Li et al. 2014). Heme oxygenase 1 (HO-1) has been recognized as a cardioprotective agent because of its ability to exert anti-inflammatory, antiproliferative, anti-apoptotic, and antioxidant activities in the cardiovascular system (Ali et al. 2007). Statins can affect HO-1 expression via transcriptional and post-transcriptional pathways. Activation of the HO-1 promoter in ECs significantly increased after 24 h of atorvastatin treatment, and this pathway was mediated by KLF2 (Ali et al. 2007). Atorvastatin does not directly affect the HO-1 gene, but influences its transcription via the KLF2-related pathway. However, atorvastatin does not slow down the rapid breakdown of HO-1 mRNA (Ali et al. 2007). This is in contrast with the findings of subsequent experimental studies. Simvastatin significantly lengthened the half-life and enhanced the stability of HO-1 mRNA in ECs, mostly via the PI3K/Akt signaling pathway. This indicates that simvastatin primarily affects the expression of HO-1 after transcription (Hinkelmann et al. 2010). These varying outcomes may be attributed to the diverse variety of statins used. There may be differences in the potency of the different classes of statins to produce pleiotropic effects. HO-1 expression was upregulated in the above studies in response to statins, which enhanced EC resistance to oxidative stress.

NO is also involved in the EC antioxidant stress response. Ota et al. (2010) found that statins inhibit EC oxidative stress-induced cellular senescence by increasing the production of eNOS, sirtuin (SIRT) 1, and catalase via the Akt-dependent pathway. NO from eNOS interacts directly with ROS and affects ROS-metabolizing enzymes by activating intracellular ROS (Haendeler et al. 2004). NO can engage in S-nitrosylation with reactive cysteine SH groups, which is the mechanism by which NO participates in redox-related signaling pathways (Stamler et al. 2001). S-nitrosylation of thioredoxin-1 (TRX)-related sites in ECs is also mediated by NO and can activate the redox-regulatory activity of TRX through S-nitrosylation of NO (Haendeler et al. 2002, p. 69). Haendeler et al. (2004) discovered that statins elevate the S-nitrosylation of TRX at cysteine 69, boosting TRX redox-regulatory function and leading to a decrease in ROS in ECs. Moreover, all the effects of statins on the redox system were reversed by mevalonate, suggesting that the antioxidant effects of statins rely on the inhibition of HMG-CoA reductase as well as the pleiotropic effects of statins (Haendeler et al. 2004). According to the above studies, statins inhibit NADPH oxidase activity and upregulate HO-1 expression and NO production, exerting antioxidative stress effects in ECs.

#### Anti-inflammation

EC inflammation is important in vascular diseases. Vascular ECs stimulated by different disease factors secrete different cytokines and express different combinations of adhesion molecules to promote inflammation, which increases leukocyte adhesion, transendothelial migration, and vascular leakage and promotes thrombosis (Wu et al. 2013). Therefore, anti-inflammation is one way to treat endothelial dysfunction and protect EC-associated barriers.

After ischemic stroke, the proinflammatory process causes injured neurons and glial cells to produce cytokines, such as TNF-α or IL-1 (Amantea et al. 2009), resulting in increased production and release of EC adhesion molecules (including ICAM-1, VCAM-1, E-selectin, and P-selectin). These adhesion molecules facilitate the attraction of circulating inflammatory cells and their movement to the ischemic area, leading to enhanced inflammatory reaction (Frijns and Kappelle 2002). Statins can decrease the expression of adhesion molecules via the classical NF-κB pathway to exert a protective effect on the BBB (Dichtl et al. 2003). In a model of transient MCAO in obese mice, rosuvastatin significantly attenuated ICAM-1 mRNA levels in brain tissue in vivo (Mayanagi et al. 2008). In in vitro experiments, atorvastatin pretreatment significantly inhibited high mobility group box-1 protein-induced vascular EC activation through modulation of ICAM-1, E-selectin, and TLR4 expression as well as NF-kB activation, suggesting that the potential protective effects of statins are partly mediated by modulation of the TLR4/NF-kB signaling pathway (Yang et al. 2010). Furthermore, statins effectively decrease the NLRP3 inflammasome in ECs by inhibiting NF-κB binding to the NLRP3 promoter, a process mediated by activation of NF-κB upstream of the nuclear pregnancy X receptor (PXR) (Wang et al. 2017). This suggests that the PXR/NF-κB/NLRP3 signaling pathway may also be a target for the anti-inflammatory effects of statins. The regulation of statins by VCAM-1 is also associated with NF-κB. Ohnesorge et al. (2010) demonstrated that KLF4 could prevent TNF-induced VCAM-1 expression in cultured ECs by obstructing the interaction between NF-kB and the VCAM-1 promoter. Additional mechanistic research indicates that fluvastatin hinders inflammation-induced activation of VCAM-1 by promoting the production of KLF4, which prevents the attachment of NF-kB to the VCAM-1 promoter in ECs (Yoshida et al. 2016). Extracellular-signal-regulated kinase 5 (ERK5) also plays an important role in the statin-induced inhibition of endothelial ICAM-1 and VCAM-1. ECs treated with XMD8-92, an ERK5 inhibitor, and transfected with ERK5 siRNA demonstrated that ERK5 inhibition abolished the suppression of VCAM-1 and ICAM-1 production triggered by TNF-α (Wu et al. 2013).

Besides adhesion molecules, statins regulate other inflammatory factors in ECs. Previous research has shown that statins can also suppress the production of MCP-1, IP-10, and IL-8 by blocking the NF-κB-related pathway (Fruscella et al. 2000). Simvastatin reduced the mRNA levels of the chemokines MCP-1, MIP-1β, and RANTES, as well as the chemokine receptor CCR4 in ECs. This effect is achieved through the effect of statins on RhoA geranylgeranylation, which influences NF-κB activity (Veillard et al. 2006). Briefly, the anti-inflammatory effects of statins in ECs are primarily mediated by the NF-κB-related signaling pathway.

#### Angiogenesis

The induction of angiogenesis is important for the recovery of neurological function and the remodeling of BBB integrity in ischemic stroke (Kanazawa et al. 2019). In many animal models of ischemic stroke, statins can enhance angiogenesis by boosting capillary density and perfusion rates in the microvascular system, aiding the restoration of vascular homeostasis (Kureishi et al. 2000; Izumi et al. 2009). Numerous studies have been conducted on the impact of statins on angiogenesis; however, the results are inconsistent. Some studies have shown that statins have proangiogenic effects, whereas others have found them to have anti-angiogenic effects (Zhang et al. 2007). The current consensus is that the angiogenic effects of statins are contingent upon their dosage (Weis et al. 2002).

Low doses of statins stimulate angiogenesis in various animal models, and the mechanism correlates with the expression of eNOS and VEGF (Zahedipour et al. 2022a). Statins can stimulate post-transcriptional phosphorylation of eNOS through activation of the PI3K/Akt and MAPK signaling pathways, which activates the VEGF-mediated migration of mature ECs and subsequent stimulation of angiogenesis (Ahmadi et al. 2023). Previous studies have indicated that simvastatin upregulates VEGF expression in ECs by increasing the protein level of the transcription factor insulin gene enhancer protein 1 (ISL1), which affects the translation process of ISL1 (Liang et al. 2020). This effect is also likely due to the activation of AMPK by simvastatin in ECs and inhibition of proteasomal activity, which inhibits proteasomal degradation and thus stabilizes the ISL1 protein (Liang et al. 2020). In addition, the regulation of angiogenesis by statins may be independent of VEGF activation. Endothelial Notch1 is crucial for vasculature remodeling during embryonic development (Limbourg et al. 2005) and angiogenesis in the ischemic limb of adults (Takeshita et al. 2007). Simvastatin has been shown to enhance the expression of Notch-related proteins in ECs in a rat stroke model (Zacharek et al. 2009). Mechanistic studies have suggested that pitavastatin activate γ-secretase and Notch1 signaling through the PI3K/Akt pathway to enhance the proangiogenic activity of ECs, while blocking or reducing the expression of Notch1 eliminated this effect (Kikuchi et al. 2011). The proangiogenic effects of Notch1 induced by pitavastatin do not affect VEGF expression, indicating that VEGF induction is not part of statin-induced Notch signaling activation (Kikuchi et al. 2011). Forkhead box O (FOXO) 1 is abundant in adult ECs, and its increased expression hinders EC migration and angiogenesis (Potente et al. 2005). Statins have been demonstrated to protect ECs by reducing the interaction between FOXO1 and KLF2 promoter region (Lee et al. 2013, p. 2). Immunoprecipitation has also demonstrated that atorvastatin can enhance the binding of S-phase kinase associated protein 2 to FOXO1, resulting in FOXO1 ubiquitination and destruction (Park et al. 2018). These results suggest that FOXO1 may be a target for the angiogenic effect of statins.

Previous studies have suggested that low-dose statins predominantly exert a cholesterol-lowering effect, whereas high-dose statins have more pronounced pleiotropic effects, significantly reducing FPP and GGPP synthesis, which leads to reduced EC proliferation and migration (Zahedipour et al. 2022b). However, there is a lack of large randomized controlled trials demonstrating whether the recommended statin dose for ischemic stroke has a pro- or anti-angiogenic effect on cerebrovascular ECs. High-dose statins did not affect eNOS upregulation, and GGPP/RhoA pathway inhibition promoted eNOS expression (Zahedipour et al. 2022a). This suggests that statins consistently produce a protective effect on ECs, regardless of dose. Furthermore, using the biphasic effects of statins may lead to new ideas for the wider application of statins.

#### Tight junction proteins and others

Statins can also directly regulate interendothelial TJPs and exert a protective effect on the BBB. Morofuji et al. (2010) conducted a detailed study of statins that regulate claudin-5 expression in ECs. They found that pitavastatin significantly increased transendothelial electrical resistance, which is an indicator of the tightness of the interendothelial tight junction (TJ) barrier. Immunocytochemical and western blotting analyses showed that the protein levels of claudin-5 were significantly elevated under pitavastatin conditions, and pitavastatin also translocated claudin-5 from the cytoplasm to the plasma membrane, increasing functional TJPs in the BBB (Morofuji et al. 2010). In addition, ZO-1 expression was regulated by statins. Simvastatin can activate small GTP-binding proteins, which activate MEKK3, MEK5, and ERK5. ERK5 then translocates to the plasma membrane and interacts with ZO-1, regulating TJ formation and reducing EC permeability to maintain BBB integrity (Wilkinson et al. 2018). Thus, statins can also modulate TJPs in ECs, affecting BBB integrity, which is one way statins directly modulate BBB integrity. In addition, statins also maintain BBB integrity by promoting the stability of the EC cytoskeleton. Fibronectin and α5β1 integrin are important components of the endothelial cytoskeleton, and statins have been found to block the reduction of fibronectin and α5β1 integrin by inhibiting the RhoA/PKC signaling pathway, thus maintaining BBB integrity (Chang et al. 2012).

Besides the pathways described above, statins can downregulate endothelin-1 (ET-1) expression in ECs and improve CBF. Hernandez-Perera et al. (1998) found that atorvastatin and simvastatin inhibited pre-proET-1 mRNA expression in a concentration- and time-dependent manner and reduced the immunoreactivity levels of ET-1. In addition, cerivastatin can induce enhanced phosphorylation of eNOS through the PI3K /Akt pathway, followed by an increase in NO production, which may lead to inhibition of ET-1 production (Ohkita et al. 2006). The interaction between NO and ET-1 also suggests that the balance between the two plays an important role in vascular homeostasis. The regulation of the interaction between NO and ET-1 is a potential target for treating vascular disorders. Statins, due to their lipid-lowering and pleiotropic properties, are important in this context.

Statins can exert their pleiotropic effects by improving EC function, attenuating oxidative stress, anti-inflammation, promoting angiogenesis, and regulating TJPs, thus participating in the maintenance of BBB integrity and function.

### Microglia

In recent decades, microglia have gained recognition for their crucial role in various functions, such as immunity, homeostasis, trophic support, neurogenesis, synaptogenesis, debris clearance, neurological recovery, protection against superoxide radicals, and self-renewal. They are now considered promising targets for therapeutic interventions in CNS-related disorders (Bagheri et al. 2020). As resident immune cells of the CNS, microglia are extremely sensitive to environmental changes, respond rapidly to external insults, and are involved in the maintenance and reestablishment of CNS homeostasis. However, the phenotype of microglia is highly plastic and their response to CNS injury is dynamic; therefore, their phenotype changes over time during the course of the disease to perform different functions (Gullotta et al. 2023). Reactive microglia in the acute phase of injury can exert protective functions; however, the persistence of ongoing inflammation can transform them into a deleterious phenotype that impairs neurons and induces BBB damage (Haruwaka et al. 2019). Most previous studies have classified activated microglia into two phenotypes. Proinflammatory microglia primarily release proinflammatory cytokines (IL-1α, IL-1β, TNF-α), chemokines (CCL2, CCL5, CXCL1, and MIP-1), and proteases (such as MMPs and ROS), which secrete factors that can orchestrate the immune response, but prolonged action leads to BBB dysfunction. In contrast, anti-inflammatory microglia mainly secrete molecules (such as TGFβ-1 and IL-10) that promote tissue healing and inhibit inflammation, which antagonizes the effects of proinflammatory microglia and plays a protective role in the BBB (Gullotta et al. 2023).

**Fig. 5:**
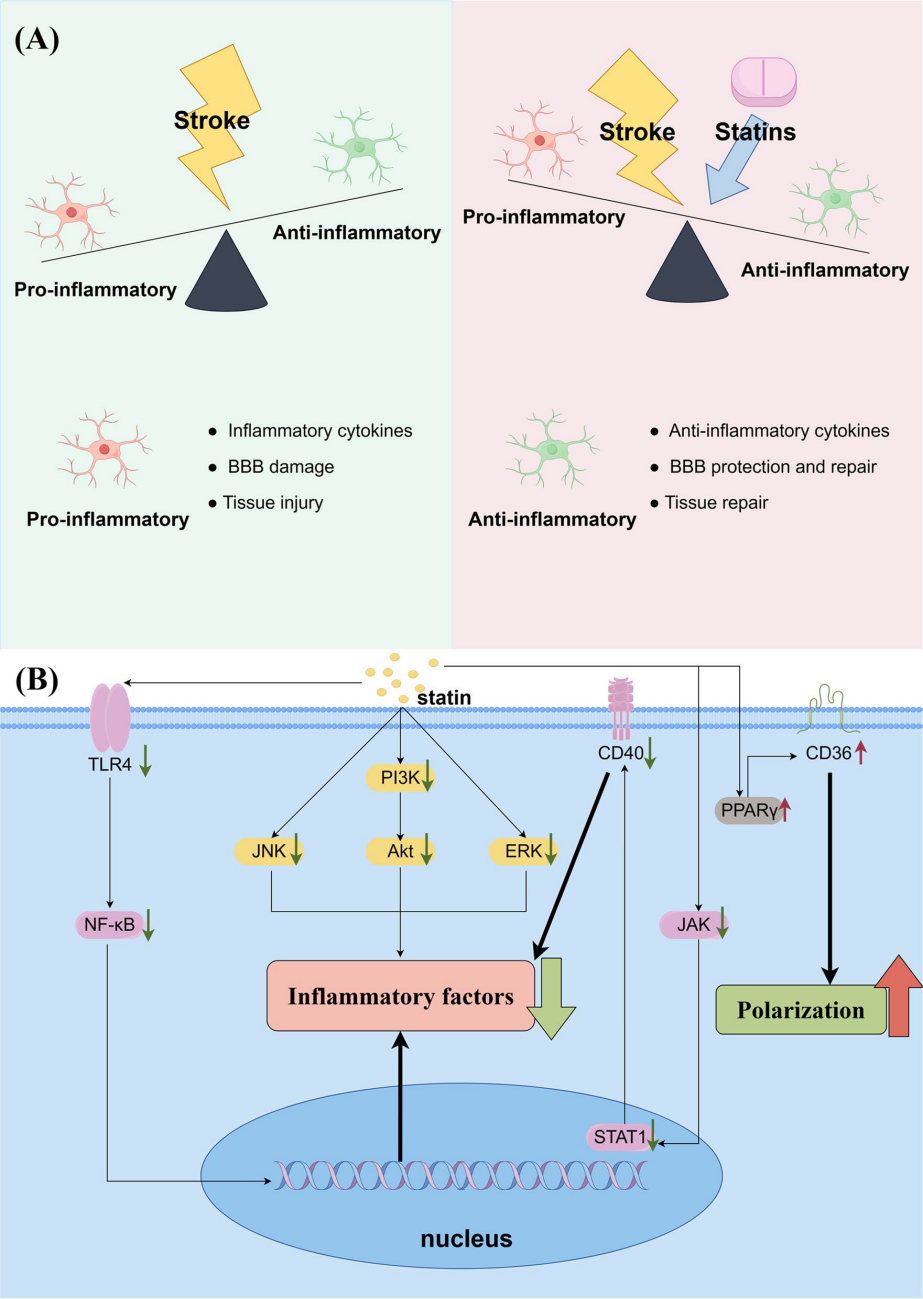
Pleiotropic effects and mechanisms of statins in microglia. **A** Influence of stroke and statins on the microglial phenotype (**B**). Mechanisms of pleiotropic effects of statins in microglia

Statins affect the function of microglia mainly from two aspects: secretion of inflammatory factors and polarization of microglia (Fig. 5).

#### Secretion of inflammatory factors

During ischemia, dying neurons release various molecules that activate microglia, leading to the release of proinflammatory cytokines. This induces a local sterile immune response, which is associated with BBB dysfunction after ischemic stroke (Candelario-Jalil et al. 2022). Accordingly, regulating the release of inflammatory cytokines and controlling inflammatory responses are important pathways for maintaining BBB function.

In microglia, NF-κB activation increases the expression of proinflammatory genes, including inducible nitric oxide synthase (iNOS), TNF-α, and IL-6. In in vivo experiments on intracranial hemorrhage (Ewen et al. 2013) and traumatic brain injury (Xu et al. 2017), atorvastatin decreased the expression of proinflammatory factors, such as IFN-γ and IL-6, and increased the protein levels of anti-inflammatory factors, such as TGF-β1 and IL-10. Han et al. (2018) found that atorvastatin reduced the release of proinflammatory factors mainly through inhibition of the TLR4/TRAF6/NF-κB pathway in microglia. Zi et al. (2021) also discovered that rosuvastatin can suppress the expression of NF-κB. ERK is a key regulatory factor for the activation, migration, and production of the cytokines TNF-α and IL-6 by microglial (Manickavasagam and Oyewumi 2019). Manickavasagam et al. (2019) found that ERK inhibitors significantly inhibited the inflammatory response induced by lipopolysaccharide (LPS) stimulation, and simvastatin significantly inhibited the LPS-induced increase in TNF-α and IL-6 levels in microglia. However, the inhibitory effect of simvastatin was significantly greater than that of ERK inhibitors alone, suggesting that statins may also inhibit the secretion of inflammatory cytokines by microglia through other mechanisms. Beyond the ERK pathway, simvastatin also inhibits the secretion of inflammatory cytokines by microglia through the JNK and Akt pathways (Manickavasagam and Oyewumi 2019).

Besides conventional signaling pathways, CD40 is also targeted by statins in microglia for its anti-inflammatory effects. CD40 plays an important role in microglial inflammatory signaling and regulation of microglial function (Tan et al. 1999). Townsend et al. (2004) found that lovastatin reduced pro-inflammation and the release of proinflammatory factors by inhibiting CD40 expression in microglia. A mechanistic study by Townsend et al. (2004) revealed that lovastatin directly and rapidly inhibited JAK/STAT1 phosphorylation and reduced CD40 mRNA levels. This suggests that statins downregulate microglial CD40 expression through inhibition of the JAK/STAT1 signaling pathway, which produces anti-inflammatory effects. Statins also chronically modulate the JAK/STAT1 pathway. Previous research has shown that statins stimulate the production of suppressor of cytokine secretion (SOCS), which then functions as inhibitors of the JAK/STAT1 pathway by either blocking JAK tyrosine kinases or preventing STAT1 factors from binding to the cytoplasmic structural domain of the receptor (Huang et al. 2003). Similarly, Nakamichi et al. (2006) found that simvastatin inhibited the expression of CCL5 in microglia by downregulating the JAK/STAT1 pathway, which is a major chemoattractant for inflammatory cells.

#### Microglia polarization

Statins participate in BBB functional maintenance and structural repair by promoting microglial polarization. Atorvastatin treatment attenuates microglial activation following traumatic brain injury. This led to a notable reduction in the mRNA levels of proinflammatory gene markers (MCP-1, iNOS, and CD11b) while causing a large increase in the expression of anti-inflammatory gene markers (Arg1, Ym1, and CD206) (Xu et al. 2017). In addition, Tregs promote microglial polarization toward an anti-inflammatory phenotype (Xie et al. 2015a). Atorvastatin influences Tregs in peripheral tissues and aids their migration to the brain following ischemic stroke, which encourages the transformation of microglia into an anti-inflammatory phenotype (Rodríguez-Perea et al. 2017). Furthermore, an in vivo study on ischemic stroke used double immunofluorescence staining to analyze the microglial marker Iba-1 and its proinflammatory phenotype marker CD16. The ratio of CD16-positive cells to Iba-1-positive cells was significantly lower in the atorvastatin group than in the MCAO group, suggesting a decrease in microglia with a proinflammatory phenotype (Zhang et al. 2021). In vivo and in vitro studies have also shown that simvastatin can promote anti-inflammatory effects in microglia by stimulating the expression of PPARγ, one pathway by which statins promote microglial polarization (Wang et al. 2018).

Unfortunately, there is a lack of direct evidence regarding the specific mechanism of action of statins on microglia. However, both microglia and macrophages are derived from the mononuclear phagocyte system, suggesting similarities in some of their biological properties. In clinical studies (Zhang et al. 2008) and in vivo studies in animals (Jo et al. 2005), statins significantly increased SOCS3 and SOCS7 expression in monocytes, inhibiting the release of proinflammatory cytokines. In addition, statin-induced SOCS3 expression in monocytes also inhibits the phosphorylation of STAT1 and STAT3, which reduces the release of IL-6 and IL-23 from monocytes (Zhang et al. 2008). These may also be the pathways through which statins regulate microglia. In conclusion, both neuroinflammation involving microglia and microglial phenotypes can be affected by statins.

### Astrocytes

Basement membrane proteins in the BBB are mainly secreted by localized astrocytes and, together with astrocyte endfeet, almost encircle the cerebrovascular system, making the maintenance of BBB integrity by astrocytes self-evident (Manu et al. 2023). After CNS damage, astrocytes undergo functional polarization, which can increase the expression of factors that restore BBB integrity. However, in many CNS disorders, the production of substances that enhance BBB permeability is heightened in responsive astrocytes, potentially worsening the tissue damage (Manu et al. 2023). During ischemic stroke, astrocytes become reactive and produce several proinflammatory cytokines, such as TNF-α, IL-1β, IL-6, and IFN-γ, in response to ischemia. These proinflammatory cytokines can act in an autocrine/paracrine manner, which leads to amplified secretion, resulting in sustained astrocyte proliferation and neurotoxicity (Trendelenburg and Dirnagl 2005).

Most existing studies on the effects of statins have focused on the cytokine secretory properties of astrocytes. Under ischemic/hypoxic conditions, astrocytes can become excessively activated by neurotoxic substances or proinflammatory factors. These activated astrocytes release cytokines and chemokines that stimulate the movement of lymphocytes through the BBB and increase BBB permeability. After treatment with atorvastatin, astrocyte activity was significantly inhibited and BBB permeability was markedly reduced (Kho et al. 2021). In a study on intracranial hemorrhage, the levels of cytokines MCP-1, IL-8, and RANTES were notably reduced when treated with statins. This effect is likely due to the anti-inflammatory properties of statins, which inhibit the release of cytokines from astrocytes (Yang et al. 2013a). Statins also modulate the production of NO by iNOS, and its oxidative byproduct peroxynitrite in astrocytes is believed to be responsible for neuronal degeneration after ischemia and in many CNS disorders, including Alzheimer’s disease (AD), Parkinson’s disease (PD), tumors, and trauma (Schmeer et al. 2006). Lovastatin has been shown to inhibit cytokine-mediated upregulation of iNOS and subsequent NO production in rat astrocytes (Pahan et al. 1997), suggesting that statins may inhibit the inflammatory response and secondary injury after acute ischemia.

Astrocytes stimulated after cerebral ischemia–reperfusion secrete MMP-9, causing degradation of the extracellular matrix, which exacerbates BBB (Wang et al. 2006). Kim et al. (2020) found that statins block the RhoA/ROCK pathway and decrease TGF-β2 production in astrocytes to decrease the levels of MMP-2 and MMP-9, which reduce extracellular matrix damage and protect the integrity of the BBB. Zhang et al. (2005a) discovered that combining atorvastatin with tPA decreased MMP-9 levels and extended the time frame for thrombolysis in a rat model of thrombus formation. Wang et al. (2006) found that the improvement in tPA-induced MMP-9 by simvastatin was mediated by the Rho pathway. Thus, besides affecting the secretion of cytokines by astrocytes, statins can maintain BBB integrity by inhibiting the Rho pathway and reducing MMP secretion.

Unfortunately, only few studies have elaborated on the specific mechanisms by which statins affect astrocyte functions. However, we hypothesized targets for the possible role of statins in protecting the BBB from some statin trials in CNS disorders.

Astrocytes can activate NF-kB to trigger CNS inflammatory responses and BBB destruction (Manu et al. 2023). Meanwhile, as astrocytes are "tightly connected" to cerebrovascular ECs, the activation of astrocyte NF-κB also affects the expression of TJPs and increases endothelial inflammatory responses and oxidative stress, which causes changes in BBB permeability (Manu et al. 2023). Morishita et al. (2014) found that simvastatin effectively decreases TNF-α-induced NF-κB activation in cultured optic nerve astrocytes, suppressing neurological inflammation. It is reasonable to hypothesize that the anti-inflammatory effects of statins in the CNS may be partially mediated by inhibition of astrocyte NF-κB activation, thus reducing astrocyte-dependent endothelial dysfunction. STAT3 is also involved in astrocyte activation. Blocking the JAK/STAT3 pathway reduces the mRNA levels of IL-6, IL-1b, IL-4, and VEGF in cell cultures (Wang et al. 2012). However, März et al. (2007) obtained controversial results. Statins do not activate classical signaling pathways, including STAT3, implying that statins may affect astrocyte activation through other pathways. Further studies must confirm these findings.

Although there is relatively little evidence that statins directly modulate astrocytes in stroke, in studies that have been conducted, statins affect the integrity of the BBB by modulating astrocyte function.

### Pericytes

Pericytes are cells that exist at various intervals along the capillary wall. The microvasculature of the CNS is thought to have a higher density of pericytes than peripheral organs, suggesting that pericytes are of special importance in the CNS (Mäe et al. 2021). Previous studies have shown that pericytes are important for vascularization, maintenance of BBB integrity, and regulation of CBF (Attwell et al. 2016). Pericytes play a role in maintaining the integrity of the BBB in at least two ways: by regulating the pattern of BBB-specific gene expression in ECs and by inducing polarization of the endfeet of perivascular astrocytes in the CNS (Armulik et al. 2010).

As mentioned for ECs, statins induce angiogenesis, and this effect is dose dependent and biphasic (Weis et al. 2002). Most previous studies concluded that statins mainly regulate angiogenesis by modulating the function of ECs to maintain the stability of cerebral blood vessels and the permeability of the BBB. However, recent studies have found that pericytes are also involved in the angiogenesis-promoting effects of statins (Armulik et al. 2010), and a large part of the regulation of BBB function by pericytes is determined by the signaling between pericytes and ECs (Yang et al. 2021). Yang et al. (2021) founded that CX43 expression was elevated in pericytes surrounding new blood vessels, and atorvastatin boosted CX43 protein levels even further. Pericytes and ECs share a basement membrane and establish direct synaptic-like connections via N-cadherin and CX43. These connections facilitate exchange between pericytes and ECs. Disruption of the interaction between CX43 and ZO-1 results in high BBB permeability (Yang et al. 2021). In addition, neural-glial antigen 2 (NG2) was highly expressed in pericytes located in newly formed blood vessels surrounding the area of tissue damage in rats after stroke and was associated with an increase in the levels of VEGF and TJPs (ZO-1 and occludin) in the ECs. Atorvastatin further increased NG2 protein expression in ischemic tissues (Yang et al. 2013b). NG2 is an established early marker of activated pericytes and has been used to identify proliferating pericytes in angiogenic microvascular systems (Virgintino et al. 2007). Thus, atorvastatin not only promoted the expression of pericyte CX43 and enhanced the connection between pericytes and ECs, but also increased the level of pericyte NG2 protein and activated pericytes, which promoted the proliferation of ECs and the synthesis of TJPs. Similarly, Baganha et al. (2021) found that atorvastatin inhibited the release of angiopoietin-2 (ANGPT2) from ECs and restored pericyte Tie2 activation, which increased the activation of Akt signaling in pericytes. Studies have demonstrated that ANGPT2 produced by ECs can hinder Tie2 signaling in pericytes and decrease Akt activation, leading to increased BBB permeability (Teichert et al. 2017). Thus, statins can affect pericytes and signaling between pericytes and EC to affect the BBB. Okamoto et al. (2002) found that pericyte apoptosis could also attenuate the restriction of EC growth. However, the in vitro effects of statins on pericyte apoptosis are unlikely to occur in human patients taking normal doses, and there is no definitive experimental evidence to prove whether statins activate pericyte regulatory functions or promote pericyte apoptosis in the maintenance and repair of BBB integrity. Statins can protect the BBB by affecting signaling between pericytes and ECs.

Another pathway by which pericytes regulate BBB function is by affecting the polarization of astrocyte endfeet. Analysis of pericyte and astrocyte gene array data revealed that several astrocyte markers were downregulated in the pericyte-deficient brain microvascular segments. Polarized astrocyte endfeet-associated markers were normally expressed and distributed in the micro cerebral vessels of the control group. However, in pericyte-deficient mutants, these markers are markedly reduced, and astrocyte endfeet are abnormally polarized (Armulik et al. 2010). Although there is substantial experimental evidence for statin modulation in astrocytes, further studies are needed to determine whether statins can modulate astrocyte function via pericytes.

Nevertheless, in the available studies, statins can exert a role in maintaining BBB integrity and promoting BBB repair by modulating pericyte function.

### Neurons

**Fig. 6:**
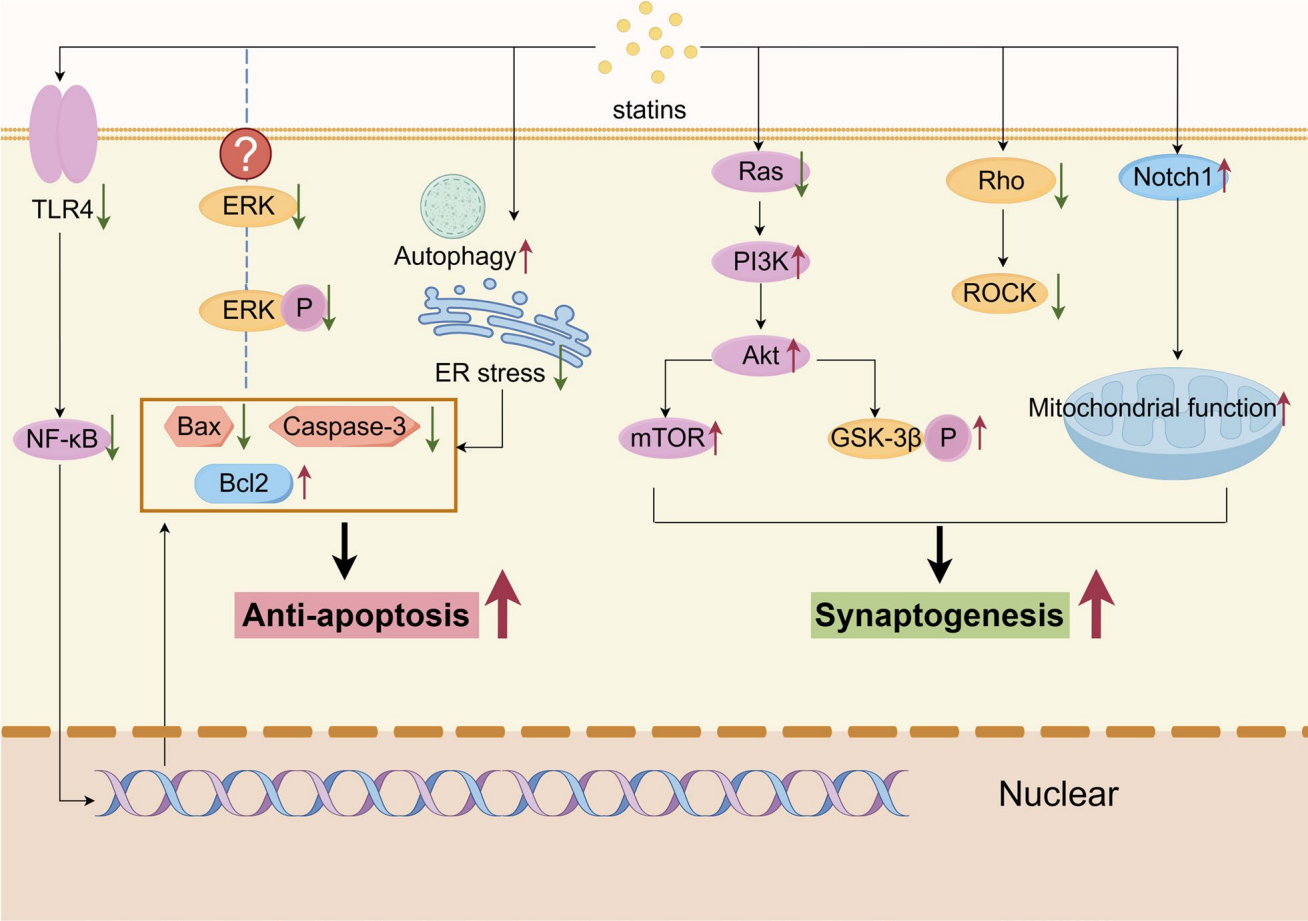
Pleiotropic effects and mechanisms of statins in neurons. Statins affect the expression of apoptosis and anti-apoptosis related proteins through various pathways resulting in anti-apoptotic effects. The pleiotropic effects of statins in neurons also modulate synaptogenesis

Neurons play the role of "commander" in the NVU. Because of their high sensitivity to the surrounding environment and their ability to establish connections and transmit signals to the surrounding cells, when neurons detect changes in the concentration of nutrients, oxygen, and metabolites in the surrounding environment, they can transmit the information of such changes as electrical and chemical signals to the surrounding cells to regulate the function of the other cells in the NVU (Muoio et al. 2014). As a component of the NVU, neurons may be potential targets for the NVU to maintain BBB integrity and promote BBB repair. Regarding most studies of neuroprotective agents in ischemic stroke and considering the pleiotropic effects of statins on neurons (Gutiérrez-Vargas et al. 2015), we concluded that statins may affect the BBB via neurons through two pathways: (1) anti-apoptosis: reducing neuronal death to maintain BBB integrity and (2) synaptogenesis: promoting synaptogenesis to facilitate neuronal connections to the BBB (Fig. 6).

#### Anti-apoptosis

Neuronal apoptosis-related signaling pathways are among the targets of most neuroprotective agents, and statins are no exception. In vivo and in vitro studies have shown that statins reduce neuronal death in various neurological disorders (Johnson-Anuna et al. 2007; Kho et al. 2021). Under OGD conditions, the expression of pro-apoptotic proteins (Bax and caspase-3) was significantly upregulated in hippocampal neurons, whereas the expression of the anti-apoptosis-related protein (Bcl-2) was suppressed. After the addition of atorvastatin, which inhibits the TLR4/TRAF6/NF-κB pathway, the expression of Bax and caspase-3 is suppressed, and the expression of Bcl-2 is significantly upregulated, increasing the rate of hippocampal neuronal survival (Han et al. 2018). Another in vivo study suggested that simvastatin could also upregulate Bcl-2 gene expression at the transcriptional level, and long-term treatment with simvastatin significantly elevated Bcl-2 mRNA levels in neurons (Johnson-Anuna et al. 2007). In addition, the ERK signaling pathway induces neuronal apoptosis by regulating the expression of apoptosis-related factors after stroke (Li et al. 2014). Hu et al. (2018) discovered that ERK expression and phosphorylation levels in hippocampal neurons were significantly reduced after simvastatin treatment, and caspase-3 was significantly lower and Bcl-2 was significantly higher in hippocampal neurons than in the PBS-treated group.

Statins are neuroprotective in ECs by improving EC function and maintaining BBB integrity through the activation of the ERK signaling pathway. This is diametrically opposed to the regulatory mechanisms of statins in neurons. In addition, ERK is differentially expressed in different parts of the brain during cerebral ischemia. pERK is expressed significantly more in the ischemic penumbra than in the ischemic core, which has led numerous researchers to believe that the elevation of pERK promotes cell survival (Li et al. 2014). Activation of ERK in ischemic brain tissue enhances the endogenous antioxidant capacity of the brain tissue, promotes the expression of neuro survival factors in the ischemic region, and upregulates the levels of phosphorylated ERK1/2 induced by ischemia (Beretta et al. 2011). Therefore, there may be differences in the mechanism of statins on different cell types in the NVU, which should be explored in future studies. Moreover, different diseases may also affect the mechanism of statins, and most current studies related to the anti-apoptotic effects of statins have focused on neurodegenerative disease models. Stroke-related models have often investigated the expression of relevant proteins in the brain tissue and are less often specific to a particular cell, which may be affected because constructing the NVU in vitro is difficult.

Besides directly affecting the expression of apoptosis-related proteins, statins reduce neuronal death through cellular autophagy and endoplasmic reticulum stress-related pathways. A previous report proposed that using statins to address deficiencies in cellular autophagy and endoplasmic reticulum stress might be a possible therapy to decrease brain damage in ischemic stroke (Tripathi et al. 2016). Zhang et al. (2018) found that autophagy was significantly increased after the autophagy-related protein LC3 upregulation in the peri-infarct region after ischemia and that it reduced the infarct size. Compared to the MCAO control group, the number of neurons was significantly increased after atorvastatin pretreatment. Varmazyar et al. (2019) found that simvastatin pretreatment increased LC3 levels in neurons while downregulating the expression of p62, consequently enhancing neuronal autophagic activity. Previous studies have demonstrated that the mevalonate pathway influences autophagy mechanisms; specifically, inhibition of HMG-CoA reductase leads to a decrease in intracellular GGPP, which promotes the initiation of autophagy (Zhang et al. 2013). This suggests that statins may enhance autophagic activity in cells by inhibiting GGPP. To further elucidate the mechanisms by which statins regulate autophagy, Carloni and Balduini (2020) conducted simvastatin preconditioning in neonatal rats, which were subsequently subjected to ischemia-hypoxia. They replicated the findings of Varmazyar et al. (2019) and additionally discovered that simvastatin pretreatment further reduced mTORC1 activity while preserving mTORC2 activity, and prevented ischemia-hypoxia-induced depletion of SIRT1. This indicates that simvastatin treatment may regulate autophagy and survival pathways by modulating the activities of mTORC1, mTORC2, and SIRT1. SIRT1 has been shown to participate in autophagy regulation, operating indirectly through the activation of AMPK and the inhibition of mTOR, which includes the deacetylation of autophagy-related proteins ATG5, ATG7, and LC3, thus further influencing autophagic mechanisms (Ghosh et al. 2010). A similar phenomenon was observed when statin treatment was administered after ischemic stroke. The protein expression levels of pAMPK, mTOR, Beclin 1, and autophagy-related protein 7 (ATG7) were reduced in the brain tissue of the simvastatin pretreated and treated groups compared to the ischemic stroke group (Ghosh et al. 2024). Furthermore, the mRNA levels of Beclin 1 and ATG7 were downregulated in both the simvastatin pretreatment and treatment groups. Additionally, this study demonstrated that simvastatin administration promoted the formation of autophagic lysosomes after ischemic stroke, evidenced by a significant increase in the co-localization of LC3B with lysosome-associated membrane protein 2 (LAMP2) and LC3B with cathepsin B (Ghosh et al. 2024). Thus, simvastatin can enhance neuronal autophagic activity following ischemic stroke by regulating the p-AMPK/LC3B/LAMP2 axis, ultimately promoting neuronal survival.

#### Synaptogenesis

The connection between neurons and various members of the NVU mainly relies on synapses; thus, inducing synapse generation and connection, as well as restoring synaptic function, are also targets of treatment in NVU reconstruction. The most crucial step in the reconstruction of neurovascular coupling is neurite outgrowth.

Previous research has indicated that pravastatin stimulates neurite outgrowth by inhibiting the RhoA signaling pathway through the reduction of geranylgeranylation (Pooler et al. 2006). Akt has been identified as a significant mediator of several features of neurite outgrowth, including elongation, branching, and caliber, besides the Rho family of proteins (Read and Gorman 2009). Jin et al. (2012) were the first to demonstrate that the PI3K/Akt pathway plays a role in atorvastatin's enhancement of neurite outgrowth and atorvastatin stimulates Akt via Ras activation, leading to increased synapse expansion in cortical neurons. In addition, Akt regulates neurogenesis mostly through its downstream mTOR pathway, as studies have demonstrated that the PI3K/Akt/mTOR signaling pathway promotes the expansion and branching of hippocampal neurons (Chen et al. 2003b). These results suggest that atorvastatin stimulates neurite outgrowth by activating the PI3K/Akt pathway and enhancing mTOR phosphorylation. Activated Akt also phosphorylates GSK-3β at the Ser9 site, inactivating its kinase activity, which enhances microtubule polymerization and dendrite outgrowth (Goold et al. 1999). Atorvastatin treatment of cultured cortical neurons increases GSK-3β phosphorylation at Ser9, and this effect is likely mediated by Akt regulation (Jin et al. 2012). Thus, besides mTOR, statins can regulate neuronal dendrite production by affecting GSK-3. Neurite outgrowth is an energy-intensive process, with mitochondria being the primary organelles responsible for energy production in cells. Mitochondria can have a significant impact on the regulation of brain plasticity, such as synapse expansion (Cheng et al. 2010). An in vitro study demonstrated that impaired mitochondrial biogenesis after ischemic stroke leads to reduced mitochondrial function, which interferes with neuroplasticity (Wang et al. 2014). However, enhancing mitochondrial function may reduce brain damage associated with ischemic stroke (Valerio et al. 2011). These findings suggest that rosuvastatin treatment reduced OGD-induced mtDNA loss in cortical neurons. The same result was obtained by He et al. (2018), who found that the inhibition of Notch1 signaling reversed the positive effects of rosuvastatin treatment on mitochondrial biosynthesis and function. Rosuvastatin promotes neurite outgrowth by activating the Notch1 signaling pathway and regulating mitochondrial function. Furthermore, in vivo experiments have revealed that simvastatin intervention (both therapeutic and preventive) can reduce the infarct volume in rat brains and facilitate the recovery of neurobehavioral and motor coordination functions. Further mechanistic studies have shown that simvastatin can improve the activities of mitochondrial complexes, reduce the production of mitochondrial ROS, and enhance mitochondrial membrane potential. These beneficial effects exhibit a dose-dependent relationship (Sarmah et al. 2023). Additionally, it has been found that atorvastatin administered via gavage to MCAO rats can inhibit neuronal apoptosis, with the mechanism related to blocking the opening of the mitochondrial permeability transition pore and the release of cytochrome C (Song et al. 2014).

Apart from promoting neurite outgrowth, statins also stabilize the structure of synapses such that they can function properly. After ischemic stroke, atorvastatin restored the levels of N-cadherin, p120 catenin, and ∝ N catenin in the cerebral cortex and hippocampus. It also enhances the association of the cadherin/catenin complex with the PSD-95 protein, which is involved in neuronal connectivity (Céspedes-Rubio et al. 2010). Furthermore, atorvastatin therapy inhibits the cytoplasmic localization of glutamate receptor subunits (NR1 and NR2B NMDA) and reinstates their interactions with PSD95 and synaptic adhesion proteins. The latter has been associated with the restoration of microtubule stability after statin treatment (Gutierrez-Vargas et al. 2014), thus allowing receptors to be transported and remain stable in the synapse. Statins also activate the PI3K/Akt pathway and Ras/ERK, both of which transduce survival signals and promote synaptic plasticity (Gutiérrez-Vargas et al. 2015). Moreover, administration of a low dose of atorvastatin 24 h after ischemic stroke effectively increased synaptophysin expression in the ischemic penumbra, leading to improved neurological outcomes. Synaptophysin, a synaptic vesicle-associated protein, serves as an indicator of presynaptic plasticity and synaptogenesis (Chen et al. 2003b). Yang et al. (2011) also demonstrated that atorvastatin and simvastatin significantly increase synaptophysin density in tissues surrounding hematomas in rats after hemorrhagic stroke.

Although many previous studies have explored the roles and pathways of neurons in the NVU components, many of the specific connections and mechanisms remain unclear. The above mechanisms by which statins affect the BBB via neurons are also the results that we have summarized and speculated from existing studies. More research is required to refine the function of neurons in the NVU.

### Challenges and difficulties in the clinical use of statins

#### Relationship between statins and intracranial hemorrhage

Due to the antiplatelet and antithrombotic effects of statins, numerous investigators have suggested that statins may increase the risk of intracranial hemorrhage in the treatment of patients who suffered a ischemic stroke (Undas et al. 2014). The Stroke Prevention by Aggressive Reduction in Cholesterol Levels (SPARCL) study (Amarenco et al. 2009) and the HPS study (Collins et al. 2003) both found that statin therapy in patients with previous ischemic stroke would increase the risk of intracranial hemorrhage, and there is even a study suggesting that patients with a history of intracranial hemorrhage should avoid statin therapy (Westover et al. 2011). Although statins have been recognized as secondary prevention drugs for stroke in several countries, the relationship between statins and intracranial hemorrhage has always been an unavoidable topic when statins are used for nervous system disease, especially stroke. However, an increasing number of studies have recently produced results contrary to those of previous studies, especially in meta-analyses of the last few years. Most studies have concluded there is no significant association between statin therapy and intracranial hemorrhage, and even if some studies have found that statins may increase the risk of intracranial hemorrhage, these have occurred in the application of high doses of statins (Table 2).

**Table 2:** Meta-analysis of the relationship between statins and intracranial hemorrhage

Author	Time	Number of studies included	Sample size included	Patient's disease type	Type of statins	Relationship with ICH	Main conclusion
Cui et al. (2022)	2022	8	10,344	AIS	Atorvastatin, simvastatin, rosuvastatin, pravastatin	Positive correlation/dose correlation	• There is no significant relationship between statin pre-treatment and ICH in patients undergoing IVT, but high-dose statins may be associated with sICH
Guo et al. (2021)	2021	22	15,000	AIS	Undifferentiated	Positive correlation	• In AIS patients receiving IVT, the use of statins before stroke is potentially associated with a higher risk of sICH
Teoh et al. (2019)	2019	15	11,576	Previous stroke	Simvastatin, rosuvastatin, pravastatin, atorvastatin	Positive correlation	• Statins can increase the risk of ICH in stroke patients
Tan et al. (2019)	2019	25	26,327	AIS	Undifferentiated	Unrelated/negative correlation	• Regardless of previous treatment with statins, receiving statins after AIS does not increase the risk of ICH; • Previously not receiving statin therapy, receiving statin therapy after AIS can reduce the incidence of ICH
Ziff et al. (2019)	2019	43	317,291	Previous stroke	Undifferentiated	Unrelated	• Statins do not increase the risk of ICH or recurrent stroke in patients with previous IS or ICH
Scheitz et al. (2016)	2016	5	8535	Previous stroke	Undifferentiated	Unrelated	• There is no significant relationship between statin use and secondary ICH in patients who have previously used statins after stroke; • For patients who have not previously used statins, using statins after stroke does not increase the risk of ICH; • The use of statins will not affect the safety of IVT
Pandit et al. (2016)	2016	7	62,204	Cardiovascular disease	Atorvastatin, simvastatin, pravastatin, rosuvastatin	Positive correlation/dose correlation	• High dose statins are positively correlated with the risk of ICH
McKinney and Kostis (2012)	2012	31	91,588	Stroke	Undifferentiated	Unrelated	• Statin therapy is not associated with ICH, and statin therapy can significantly reduce all stroke and all-cause mortality rates

*IVT* intravenous thrombolysis, *ICH* intracranial hemorrhage, *sICH* symptomatic intracranial hemorrhage, *AIS* acute ischemic stroke, *IS* ischemic stroke

There are several reasons why previous randomized controlled trials have shown conflicting results when analyzed. First, numerous studies did not differentiate patients according to treatments like thrombus extraction and intravenous thrombolysis. Second, numerous studies did not consider the patients’ history of statin therapy; patients previously treated with statins may have vascular risk factors of their own, which may also contribute to the development of intracranial hemorrhage. Furthermore, the timing of statin initiation was not clearly differentiated, including pre-stroke, in-hospital, and post-discharge statin use. In addition, compliance with statins is a major and important factor influencing studies, with over 35% of patients in several ischemic stroke studies showing poor adherence (Colivicchi et al. 2007; Kim et al. 2017; Chung et al. 2018; Vitturi and Gagliardi 2021).

In conclusion, the effect of statins on intracranial hemorrhage after stroke may need to be supported by further evidence. With the widespread use of statins, large-scale, long-term follow-up studies of non-statin users have become increasingly difficult.

### Other side effects of statins

The side effects of statins are usually associated with long-term treatment at high doses. Besides common muscular symptoms, recent studies have found that statins can increase lipoprotein (a) levels, which is an independent risk factor for cardiovascular disease and calcific aortic stenosis (Arsenault et al. 2018). Furthermore, randomized controlled trials, meta-analyses, and genetic studies have shown that treatment with statins also significantly increases the incidence of type 2 diabetes mellitus (Sattar et al. 2010), which may be related to statin activation of NLRP3 inflammatory vesicles (Henriksbo and Schertzer 2015). Some statins have also been found to affect insulin secretion through direct, indirect, or combined effects on pancreatic β-cell calcium channels (Mohammadkhani et al. 2019). Simultaneously, the lipid-lowering effects of statins cannot be ignored. Cholesterol is one of the most important components for maintaining cellular function and structural stability. In the CNS, neurons cannot synthesize enough cholesterol on their own; therefore, they need to be supplied with additional cholesterol via glial cells (Mauch et al. 2001). In contrast, the long-term application of statins may cause decreased lipoprotein delivery to glial cells, which is insufficient to maintain neuronal structural and functional stability. Sustained low levels of cholesterol have been found to reduce the levels of circulating astrocyte-derived lipoproteins, which significantly reduces neuronal membrane repair as well as the availability of lipids required for formation, maintenance, and synaptic function (Dong et al. 2009).

Optimizing the management of statin-induced side effects has emerged as a prominent clinical concern. Presently, there exist three primary strategies for mitigating statin-related adverse effects: (1) the transition to an alternative statin or the administration of a reduced dosage, with intermittent statin use also considered as an option; (2) the incorporation or substitution of alternative lipid-lowering therapeutic modalities; (3) the continuation of statin therapy while concurrently addressing symptom resolution (Mancini et al. 2011). Given the substantial benefits of statins in diminishing cardiovascular incidents, and the demonstrated cardiovascular advantages that outweigh the absolute risks associated with statin side effects (Jukema et al. 2012), the practice of switching to a different statin or employing a lower dose remains the most prevalent clinical approach. A retrospective investigation (Gadarla et al. 2008) showcased that the administration of 5 mg or 10 mg rosuvastatin on a twice-weekly basis enhances patient tolerance and maintains the lipid-lowering efficacy of statins. Additionally, extended-release fluvastatin at a dosage of 80 mg/day has been proposed for patients experiencing statin-associated myopathy (Jacobson 2008), with a lower fluvastatin dosage being advocated in a study (Jacobson 2008) and corroborated as well-tolerated in the majority of patients within a randomized controlled trial (Stein et al. 2008).

Regrettably, the majority of studies have concentrated merely on whether low-dose statin therapy can yield lipid-lowering outcomes, without delving into their pleiotropic effects. This constitutes an area that warrants exploration and is an inevitability for the future clinical application of statin pleiotropy. Furthermore, upon revisiting the comprehensive literature on the use of statins in animal models of stroke, we regret to find that further exploration of the toxic and adverse effects of statins (such as histological examination of liver and kidney tissues, blood sample analysis, etc.) has been lacking in these studies. The presumed reasons for this may encompass several factors: Firstly, the adverse effects of statins in vivo often manifest after prolonged or excessive administration, which can be difficult to accurately reflect in the constructed acute animal disease models, as many observed changes in indicators may be attributed to the disease itself. Secondly, the majority of studies have been designed with relatively short durations, with a primary focus on the efficacy of intervention strategies, thereby neglecting the safety and potential toxic effects of long-term treatment.

### Differences in the types of statins

Statins are mainly classified into two groups according to their lipophilicity: lipophilic and hydrophilic statins (Wood et al. 2014). Lipophilic statins demonstrate a superior capacity to penetrate tissues from the bloodstream, with their lipophilicity directly influencing their ability to traverse the BBB and thereby impact the pleiotropic effects of statins (Wood et al. 2014). Hydrophilic statins are more specific for tissue selection because they must enter the interior of the cell through active transport, which also means that their lipid-lowering effects are more potent than their pleiotropic effects (Irwin et al. 2020). This may be one reason for the contradictory or negative results of some pleiotropic studies. For instance, atorvastatin (lipophilic) demonstrates a superior capacity to repair BBB disruption resulting from cerebrovascular disturbances compared to pravastatin (hydrophilic) (Pallebage-Gamarallage et al. 2012). Furthermore, a study involving spontaneously hypertensive stroke-prone rats, which were chronically treated with either rosuvastatin (hydrophilic) or simvastatin (lipophilic), revealed that rosuvastatin—unlike simvastatin—exerted a beneficial effect by modulating the inflammatory processes that precede the onset of cerebral damage (Sironi et al. 2005). Clinical studies have also indicated variability in neurological outcomes associated with different types of statins (Christophe et al. 2020).

In addition, the different metabolic forms of statins are related to their pleiotropic effects. Statins are mainly as lactones and acids in the brain. The acid form of statins inhibits HMG-CoA reductase, which directly leads to a decrease in the production of mevalonate and thus to a decrease in downstream products, including cholesterol and isoprenoids, which are the main forms in which statins exert their effects. The lactone form of statins has not been shown to have an effect independent of the acid form (Wood et al. 2014). However, statins have been speculated to modulate the expression of certain genes that may be related to different metabolic forms of statins (Johnson-Anuna et al. 2005). The effect of statin type should also be considered when exploring the role of statins in CNS disorders.

### Translating statins for clinical applications

Numerous studies have reported different or even contradictory results, particularly in stroke studies (Li et al. 2014) and these differences were related to several factors. First, the results obtained from in vivo studies, animal models, in vitro studies, or cellular models may not be comparable because of the differences between the two. In vivo studies have shown that statins may also affect other cells that can transmit signals to the cells studied, thus affecting the results (Sun et al. 2018). The NVU works together to maintain its function. However, similar interfering factors may not be present in cellular models. Second, even “subtle” changes in the expression of ICAM-1 can be detected in vitro; however, in vivo, changes in its expression may not be evident in the whole tissue. In addition, there are differences between species in the pathways that regulate the expression and release of various molecules. For example, SSAO/VAP-1-dependent effects in human ECs increase the release of E-selectin and expression of P-selectin, but they occur oppositely in mice (Sun et al. 2018). These differences should be noted when translating the pleiotropic effects of statins to the clinic, and more clinical studies are needed to explore the integral effects of the pleiotropic effects of statins through various pathways in vivo so statins can be better used and promoted in the clinic.

## Conclusion

Statins, besides their lipid-lowering effects, can also regulate individual members of the NVU through pleiotropic effects, resulting in the maintenance of BBB integrity and homeostasis, ultimately producing neuroprotective effects in ischemic stroke. We summarized their effects on each cell type within the entire NVU and provided a theoretical basis for the clinical translation of the pleiotropic effects of statins. However, owing to various factors, numerous issues still need to be resolved for the wide application of statins as neuroprotective agents in the clinic, such as the type, dosage, and mode of drug delivery, which need to be explored and supplemented by more studies.

## Data Availability

No datasets were generated or analysed during the current study.

## Abbreviations

BBB: Blood–brain barrier
NVU: Neurovascular unit
HMG-CoA: 3-Hydroxy-3-methylglutaryl-coenzyme A
LDL-C: Low-density lipoprotein cholesterol
CBF: Cerebral blood flow
HPS: Heart protection study
FPP: Farnesyl pyrophosphate
GGPP: Geranylgeranyl pyrophosphate
ECs: Endothelial cells
TJPs: Tight junction proteins
NO: Nitric oxide
eNOS: Endothelial nitric oxide synthase
RNAP: RNA polymerase
FTO: Fat mass and obesity-associated protein
MCAO: Middle cerebral artery occlusion
BH4: Tetrahydrobiopterin
GCH1: GTP cyclohydrolase 1
AMPK: Adenosine monophosphate-activated protein kinase
KLF2: Kruppel-like factor 2
SR-B1: Scavenger receptor-B1
EPCs: Endothelial progenitor cells
VEGF: Vascular endothelial growth factor
NADPH: Nicotinamide adenine dinucleotide phosphate
HO-1: Heme oxygenase 1
SIRT: Sirtuin
TRX: Thioredoxin
PXR: Pregnancy X receptor
ERK5: Extracellular-signal-regulated kinase 5
ISL1: Insulin gene enhancer protein 1
TJ: Tight junction
ET-1: Endothelin-1
iNOS: Inducible nitric oxide synthase
SOCS: Suppressor of cytokine secretion
AD: Alzheimer’s disease
PD: Parkinson’s disease
NG2: Neural-glial antigen 2
ANGPT2: Angiopoietin-2
SPARCL: Aggressive reduction in cholesterol levels
ICH: Intracranial hemorrhage
